# Co-occurrence of Amyloid Goiter and Adipose Metaplasia in a Patient With History of Pulmonary Tuberculosis: A Case Report

**DOI:** 10.7759/cureus.36008

**Published:** 2023-03-11

**Authors:** Zain Ahmed Khan, Sadaf Ahmad, Rajkumar Williams, Kumaragurubaran Gnanasambandam, Mekala Lakshminarayanan

**Affiliations:** 1 Department of General Surgery, Billroth Hospitals, Chennai, IND; 2 Department of Histopathology, Apollo Cancer Centre, Chennai, IND

**Keywords:** fine needle aspiration cytology (fnac), thyroidectomy, plasma cell dyscrasias, chronic inflammatory disease, diffuse goiter, multinodular goiter, amyloidosis, pulmonary tuberculosis, adipose metaplasia, amyloid goiter

## Abstract

Amyloid goiter is described as an accumulation of amyloid, an amorphous proteinaceous material, in the thyroid gland. The deposition of amyloid is relatively common in the thyroid gland. However, a significant clinical enlargement due to amyloid accumulation and fat deposition in the thyroid stroma resulting in diffuse goiter leading to compressive symptoms is a rare phenomenon. In this report, we describe a rare case of amyloid goiter with adipose metaplasia in a 38-year-old woman with a history of pulmonary tuberculosis who presented to the outpatient department with complaints of heartburn, abdominal discomfort, and hoarseness of voice. Incidentally patient had diffused multinodular neck swelling. Preliminary blood investigations were normal. The contrast-enhanced computed tomography neck showed multiple non-enhancing lesions and a diffusely enlarged thyroid gland, causing a mass effect on the oropharynx posteriorly and minimally on the trachea. Fine needle aspiration cytology thyroid revealed thyroiditis. The patient underwent a total thyroidectomy, and histopathological examination of the specimen showed an extracellular eosinophilic amorphous substance that was positive for Congo red and showed apple-green birefringence under polarized light, and large areas of adipose metaplasia were noted, and a diagnosis was made. The amyloid involvement can result from localized primary deposition or secondary to chronic inflammatory disease. The prevalence of amyloid goiter in developed countries is due to primary amyloidosis, and in developing countries is due to secondary amyloidosis. Patients with a history of pulmonary tuberculosis commonly present with renal amyloidosis as its complication. Patients with an enlarged thyroid gland and a history of chronic inflammatory conditions or plasma cell dyscrasias should be evaluated with extreme suspicion. The correlation of tuberculosis with the subsequent development of amyloid goiter highlights the need for research in this area.

## Introduction

Amyloid goiter is described as an accumulation of amyloid, an amorphous proteinaceous material, in the thyroid gland in sufficient amounts to cause an increase in the size denotes amyloid goiter [1]. In occasional cases, the accumulation of fat coexists with the deposition of amyloid [2]. Although amyloid deposition in the thyroid is relatively common, a clinically significant enlargement due to its accumulation is rare. The discovery and understanding of the deposition of microscopic amyloid infiltration in the thyroid gland can be traced back to Carl von Rokitansky's work in 1855. Rudolf Eiselberg in 1904 described findings of a case of thyroid enlargement caused by unusual amyloid deposits and named this rare condition “Amyloid Goiter” [1,3,4]. This report presents a very rare case of amyloid goiter with adipose metaplasia in a patient with a history of pulmonary tuberculosis.

## Case presentation

A 38-year-old woman with a history of pulmonary tuberculosis at five years of age, with no known co-morbidities, presented to the outpatient department with complaints of heartburn, abdominal discomfort, and hoarseness of voice. Incidentally, the patient had diffuse neck swelling, which was insidious in onset with gradual growth over the past two years. The patient exhibited neither hypothyroidism nor hyperthyroidism symptoms.

On examination, diffuse swelling was seen on the anterior aspect of the neck, which moves with deglutition. On palpation, bilateral multinodular swellings were noted, which were firm and non-tender, with the right side more prominent than the left side. Systemic examination was unremarkable.

Blood investigations showed normal thyroid function tests, thyroglobulin antibody, and thyroid peroxidase antibody levels. Complete blood count and electrolytes were within normal range. No indicators of renal failure or proteinuria were seen on lab results, and an abdominal ultrasound suggested normal kidneys, colon, liver, and spleen. Transthoracic echocardiography and chest x-ray were normal.

**Figure 1:**
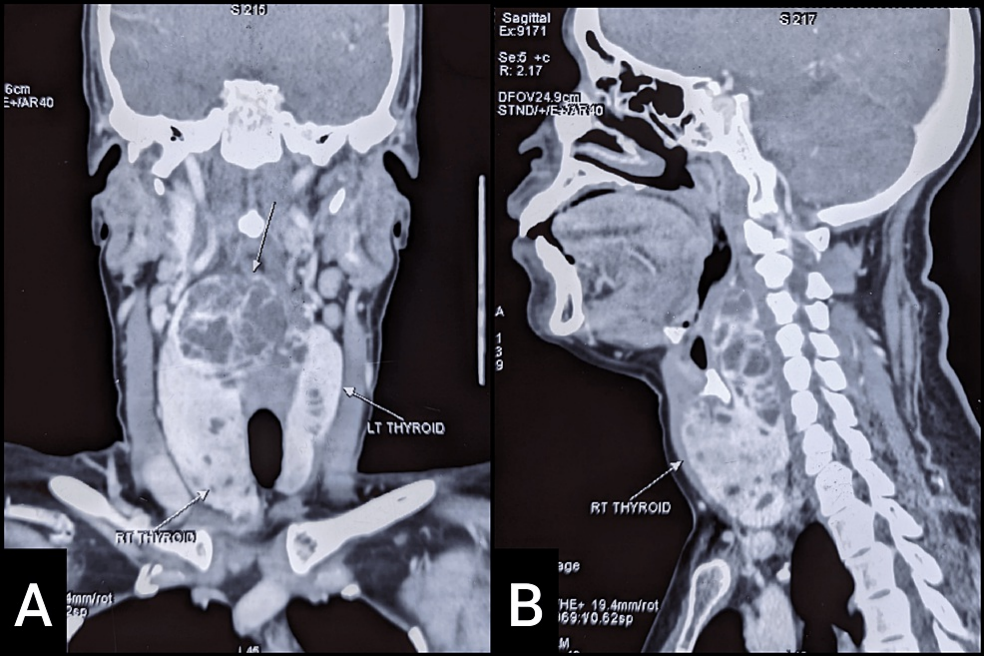
The contrast-enhanced computed tomography (CECT) of neck. (A) Coronal plane showing diffusely enlarged thyroid gland with the right lobe larger than the left lobe. (B) Sagittal plane showing enlarged right lobe of thyroid gland.

Contrast-enhanced computed tomography of the neck showed a diffusely enlarged thyroid gland with the right lobe larger than the left lobe. Multiple non-enhancing lesions were scattered throughout both lobes. There was a sizeable multiloculated lesion in the retropharyngeal space, causing a mass effect on the oropharynx posteriorly and minimally on the trachea. The largest lesion measured 4.1 cm x 2.1 cm x 3.8 cm. No vessel encasement and lymphadenopathy were noted (Figures 1A, 1B). A fine needle aspiration cytology (FNAC) thyroid revealed thyroiditis (Bethesda category II).

The patient had a total thyroidectomy. The parathyroid glands were normal in size and were preserved along with both recurrent laryngeal nerves. The peri-operative and post-operative period were uneventful. A histopathological examination of the specimen was requested.

**Figure 2:**
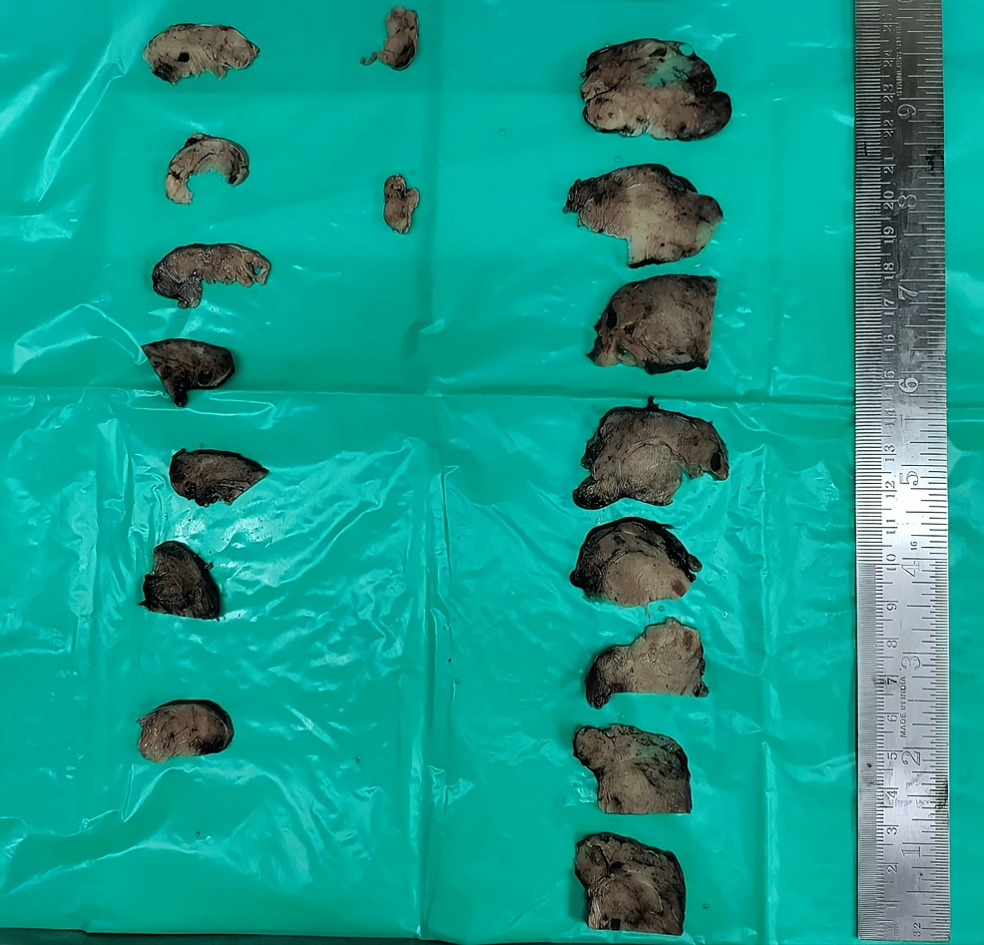
Serial sections of the resected thyroid gland.

On gross examination, the right lobe was larger than the left lobe. The right lobe measures 8.5 cm x 4.5 cm x 3 cm, and the left lobe measures 6 cm x 3 cm x 1.5 cm. Serial sectioning through both the lobes revealed tan brown to dark brown multiple nodules and focal solid tan brown to yellow areas with grey-white specks (Figure 2).

**Figure 3:**
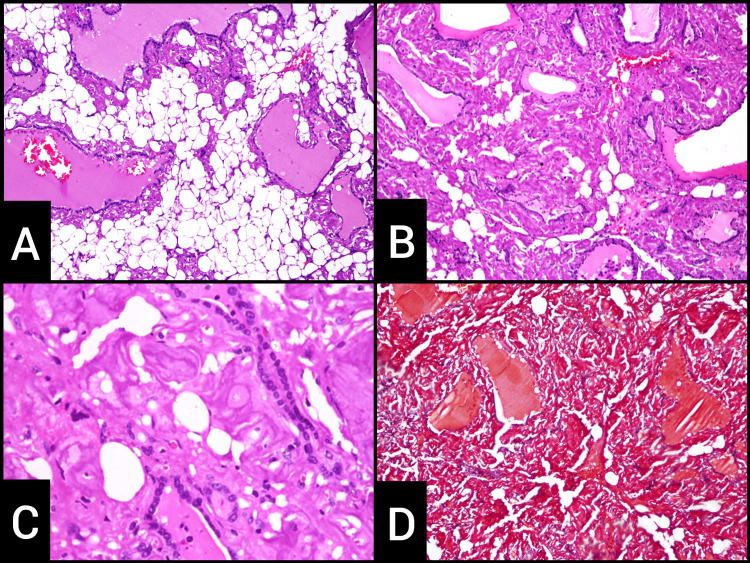
Histopathological appearance of amyloid goiter and adipose metaplasia. (A) Hematoxylin and eosin (H&E) staining of tissue showing adipose metaplasia on 100x magnification. (B) H&E staining of tissue showing adipose metaplasia and amyloid on 400x magnification. (C) H&E staining of tissue showing amyloid deposits on 400x magnification. (D) Congo red staining of tissue highlighting amyloid on 400x magnification.

Microscopic examination showed varying-sized thyroid follicles lined by flattened to cuboidal epithelium. Areas of fibro-hyalinization with extensive deposition of an extracellular eosinophilic amorphous substance were noted, which was positive by Congo red and showed apple-green birefringence under polarized light. Large areas of adipose metaplasia were noted (Figures 3A-3D). Collections of foamy and haemosiderin-laden macrophages, hemorrhage, and cholesterol clefts were seen. No atypical cells were seen.

**Figure 4:**
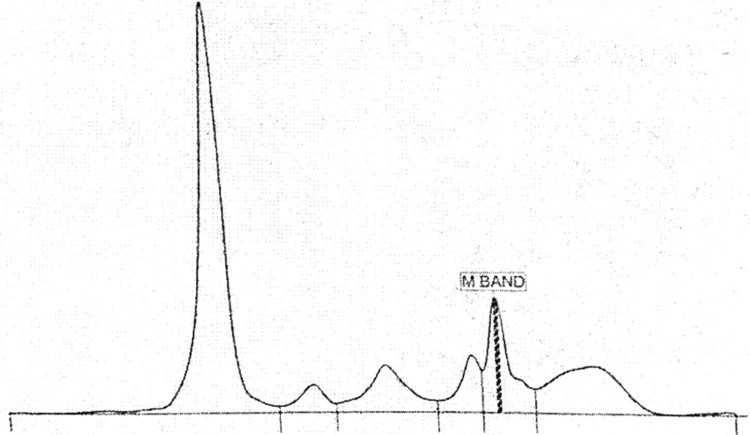
Serum protein electrophoresis curve.

In view of these findings, a diagnosis of Amyloid goiter with adipose metaplasia was made. On follow-up, several investigations were done, including CEA (1.270 ng/mL), calcitonin (<2 pg/mL), rheumatoid factor (8.6 IU/L), and serum protein electrophoresis, which showed a spike in the monoclonal band (0.26 g/dL) observed near the beta range (Figure 4), elevated serum free light chain kappa (38.03 mg/L), followed by serum immunofixation, which revealed IgG kappa monoclonal gammopathy (IgG - 1,854.4 mg/dL). The patient is currently asymptomatic and regularly followed up to delineate the disease extent.

## Discussion

Amyloid is an insoluble protein formed when soluble precursors undergo conformational change forming a protein of beta-pleated sheet configuration [1]. The amyloid involvement can result from primarily localized deposition or secondary to systemic disease [4]. 50% to 80% of individuals with medullary carcinoma of the thyroid exhibit microscopic amyloid deposition, followed by secondary amyloidosis (20%) and very rarely primary amyloidosis (15%) [1,3,4]. Most patients with amyloid goiter are euthyroid, but variations have also been reported [1]. In primary amyloidosis, there is the accumulation of major fibrillar protein amyloid L derived from IgE light chains. However, in secondary amyloidosis, there is a collection of amyloid A derived from serum amyloid A protein [3].

Amyloidosis localized to one or multiple organs, known as primary amyloidosis, is highly uncommon. Primary amyloidosis is associated with plasma cell dyscrasias. It can cause mass effects and negatively impact the normal functioning of the organs in which it gets deposited [1]. Secondary Amyloid deposition can occur due to familial Mediterranean fever, multiple myeloma, rheumatoid arthritis, and other chronic inflammatory conditions [4]. The presence of amyloid in a thyroid sample should raise concerns regarding medullary cell carcinoma and must be ruled out. The prevalence of amyloid goiter in developed countries is due to primary amyloidosis, and in developing countries due to secondary amyloidosis [1].

The deposition of insoluble proteins in amyloid goiter is due to abnormal folding, which can also lead to fat accumulation resulting in gland enlargement and causing compressive symptoms [2]. The reason for the accumulation of fat in amyloid goiter is still hypothetical. It can result from a deficiency in capillary function, causing ischemia, tissue hypoxia and triggering stromal fibroblasts to undergo metaplasia [1,5]. Amyloid goiter can present simultaneously with fat deposition [6]. This association is rare, and only a few cases have been reported. Adipocytes can be present in the normal thyroid gland in the pericapsular and subcapsular areas close to the vessels [7]. The adipose tissue deposition can also be associated with disturbed embryogenesis, as the thyroid develops from the primitive foregut [2,5,8].

FNAC is done as a thyroid nodule workup. FNACs on amyloid goiters reveal atypical follicular cells in 10% to 40% of cases [1]. The amount of fat and amyloid deposition can affect the imaging patterns in amyloid goiters [8]; hence cases can be challenging to detect. The preferred course of treatment for people with compressive symptoms is total thyroidectomy [1], but there are no definitive treatment guidelines. A post-operative histopathological examination can provide a conclusive diagnosis [1]. The recommended surgical treatment for benign multinodular goiter is total thyroidectomy to prevent a recurrence that occurs after subtotal thyroidectomy [9].

A common complication of patients with a history of pulmonary tuberculosis is renal amyloidosis, with the interval of presentation between the two ranging from six months to 42 years after infection. The hallmark of renal amyloidosis is proteinuria. In developed countries, rheumatoid arthritis is a common chronic inflammatory condition, but tuberculosis is the most common cause of secondary renal amyloidosis in developing countries [10]. Less than 100 cases of amyloid goiter have been presented in literature before 2019 [11]. In our report, we have a patient with amyloid goiter with adipose metaplasia and a history of pulmonary tuberculosis in childhood. This association is rare and provides an opportunity for further research.

## Conclusions

Our patient is one of the few rare cases presenting with amyloidosis and coexistent fat deposition. Patients with an enlarged thyroid gland and a history of chronic inflammatory conditions or plasma cell dyscrasias should be evaluated with extreme suspicion. Usually, a thyroidectomy and histological examination lead to a conclusive diagnosis. It is essential to take all possible measures to evaluate the extent of the disease and rule out any underlying secondary causes. Given the patient's history of pulmonary tuberculosis, further research is needed to explore the correlation between tuberculosis and the subsequent development of amyloid goiter. This case highlights the need for ongoing research in this area.
